# Colon-specific delivery of isoliquiritigenin by oral edible zein/caseate nanocomplex for ulcerative colitis treatment

**DOI:** 10.3389/fchem.2022.981055

**Published:** 2022-09-09

**Authors:** Meng Xiao, Shuyang Wu, Yanfen Cheng, Jiaqi Ma, Xi Luo, Liang Chang, Chen Zhang, Jianping Chen, Liang Zou, Yu You, Jinming Zhang

**Affiliations:** ^1^ State Key Laboratory of Southwestern Chinese Medicine Resources, Pharmacy School, Chengdu University of Traditional Chinese Medicine, Chengdu, China; ^2^ Li Ka Shing Faculty of Medicine, School of Chinese Medicine, The University of Hong Kong, Hong Kong, China; ^3^ Key Laboratory of Coarse Cereal Processing (Ministry of Agriculture and Rural Affairs), School of Food and Biological Engineering, Chengdu University, Chengdu, China

**Keywords:** isoliquiritigenin, zein, nanoparticle, ulcerative colitis, colon specific delivery

## Abstract

Although a natural anti-inflammatory ingredient, isoliquiritigenin (ISL), plays an effective role in ulcerative colitis (UC) treatment, a series of drawbacks still limit its clinical application, including the poor solubility, instability in gastrointestinal tract, and rapid elimination rate of ISL. Zein-based NPs display the benefits on drug loading and delivery, whereas with the poor stability. In this study, an edible nano-system composed by zein/caseinate complex was fabricated for the colon-targeting delivery of ISL, to improve its colon retention and anti-UC effects. The optimized ISL loaded zein/caseinate NPs (ISL@NPs) were prepared by single-factor design by anti-solvent precipitation method, and then characterized. The improved cellular uptake of ISL@NPs on NCM460 and RAW 264.7 cells was evaluated *in vitro*. The colon tissue permeability and retention capacity *in vivo*, and the anti-UC efficacy of ISL@NPs in DSS-induce UC were implemented. As a result, ISL@NPs with the high drug loading efficiency of 9.39% ± 0.26%, the average particle diameter of 137.32 ± 2.54 nm, exhibited the pH-sensitive stability in the different simulated gastrointestinal buffer. Compared with free ISL, ISL@NPs showed significantly higher cellular uptake ability in NCM460 and RAW 264.7 cells. Based on *in vivo* imaging system, zein/caseinate NPs showed the prolonged colonic retention and the enhanced penetration into the colonic epithelium. Finally, the oral administration of ISL@NPs could effectively alleviate the UC-related symptoms, down-regulate the production of pro-inflammatory factors, and reduce the infiltration of macrophages and neutrophils in colon tissues. In this study, an oral colon-specific nano-system, composed with the natural compound and edible materials, was developed as the promising alternatives in the prevention and treatment of UC.

## 1 Introduction

In recent years, the incidence of ulcerative colitis (UC), an idiopathic relapsing inflammatory disease affecting the colon, has been on the rise worldwide. The main UC symptoms include bloody stool, diarrhea, and colonic motility dysfunction, which greatly lower the quality of patients’ life. Even long-term progression of UC can increase the risk of colorectal cancer (Torres et al., 2012; Ungaro et al., 2017; Dong et al., 2022). The therapeutic outcomes of current agents are limited. Meanwhile, long-term administration of these chemotherapeutic agents might lead to side-effects and complications, as well as a high cost for patients (Yadav et al., 2015; Lin et al., 2019; Zhang et al., 2022a). Recently, the interventions of natural products as alternatives play the increasingly important role in the treatment and management of UC, the increasing positive examples about the anti-UC benefits of these natural medicinal extracts or compounds have been demonstrated (Liu et al., 2022). For example, polysaccharides, *Polygonati rhizome* and its polysaccharide components have great therapeutic potential for inflammatory bowel disease caused by imbalance of the oxidation-reduction microenvironment in organisms (He et al., 2022). Isoliquiritigenin (2′,4′,4-trihydroxychalcone, ISL), a flavonoid widely derived from leguminous plants like licorice, Radix Astragali, (Peng et al., 2015), and Caulis Spatholobi, (Mei et al., 2021), exhibits multiple biological and pharmacological activities such as anti-inflammatory, antioxidative, anti-tumor, and immunoregulatory effects. (Peng et al., 2015). Particularly, currently ISL has been reported to significantly ameliorate colitis *in vitro* and *in vivo* by suppressing the expression of inflammatory cytokines, such as IL-8, IL-1β, and COX-2, regulating the MAPK pathway involving in the phosphorylation of ERK1/2, p38, and AKT, and activating NF-κB pathway (Choi et al., 2016). What is more, ISL could effectively inhibit colitis-associated tumorigenesis through down-regulating M2 macrophage polarization mediated by the interplay between PGE2 and IL-6 (Zhao et al., 2014), or by modulating the intestinal microbiota (Wu et al., 2016). Therefore, the administration of ISL is likely to represent a promising strategy for the chemoprevention or treatment of UC.

Primarily, ISL as a flavonoid compound with a chalcone structure exhibits the poor water solubility, and poor gastrointestinal stability (Cao et al., 2020; Zhao et al., 2021). It was also apt to be eliminated in small intestine (Lee et al., 2013). Nanomedicine is a drug delivery system with the particle size in the range of 1–1,000 nm, which is mainly used to overcome the shortcomings of drugs easily metabolized and low bioavailability in the body, improve the accumulation capacity of drugs in target sites, and balance the relationship between the efficacy of drugs and toxic and side effects (Gao et al., 2022). Currently, the oral nano-scaled systems for colon-specific drug delivery have been conducted for UC treatment. The advantages include enhanced colon tissue accumulation capacity, the improved colonic epithelium cellular internalization, and the more sensitive drug release behavior, due to the nano-scaled size and large specific surface area (Naeem et al., 2020). Furthermore, the encapsulation of natural active compounds into nanoparticles of desired sizes can improve their solubility and biological distribution and reduce their side effects (Ganesan et al., 2021). Among these natural materials for colon delivery, zein, a water-insoluble protein from corn with high bio-safety, draws the increasing attention. Besides its biocompatibility, biodegradability and low toxicity, zein can resist the degradation resulted from gastric acid and gastrointestinal enzymes. (Wang et al., 2018). In view of those advantages, zein-based NPs have been attempted to encapsulate drugs for colon delivery. However, zein NPs are unstable and prone to aggregate in response to neutral pH (Hu and McClements, 2014). Recently, some natural polysaccharides such as pectin, fucoidan and alginate, proteins such as sodium caseinate and whey protein, or the mixture of polysaccharides and proteins, with the opposite charges, were used to increase the colloidal stability (Semenova, 2017; Dai et al., 2018b). Caseinate (Cas), a water soluble protein from milk, has been proposed to effectively stabilize zein NPs, in which zein/caseinate NPs have been used to encapsulate a variety of lipophilic chemicals (Luo et al., 2013).

However, to our knowledge, there is currently no information related to the colon-targeting delivery of zein/caseinate NPs mediated by the facile anti-solvent nano-precipitation method. Therefore, in this study zein/caseinate complex NPs were fabricated to efficiently encapsulate ISL. A series of characterization of ISL@NPs including particle distribution, stability in response to different pH values, drug encapsulation/loading efficiency, FT-IR, and XRD had been implemented. Confocal laser scanning microscopy (CLSM) was utilized to study the improved cellular internalization of ISL@NPs in colonic epithelial cells and macrophages. Finally, after oral administration of ISL@NPs, the colon-localized delivery benefit as well as the significant therapeutic outcome in DSS-induced UC mice had been observed. This work would contribute to developing an edible colon-specific delivery nanosystem for UC treatment.

## 2 Materials and methods

### 2.1 Materials

Isoliquiritigenin (ISL, purity≥98%) and sodium caseinate (Cas, purify≥90%) were purchased from Adamas (Shanghai, China). The zein was provided by Tokyo Chemical Industry (Tokyo, Japan). Dextran sulfate sodium (DSS, 36–50 kDa) was offered by Seebio Biotechnology Co., Ltd. (Shanghai, China). DiR iodide (1,1-dioctadecyl-3,3,3,3-tetramethylindotricarbocyaine iodide) was purchased from Meilunbio^®^ (Dalian, China). Cell culture supplies, including Dulbecco’s modification of Eagle’s medium Dulbecco (DMEM) medium, RPMI 1640 medium, and fetal bovine serum provided by Gibco (United States). Penicillin-streptomycin was supplied by Boster Biological Technology Co., Ltd. (United States). Trypsin was obtained from Beijing Solarbio Science and Technology Co., Ltd. (Beijing, China). Interleukin 6 (IL-6) and tumor necrosis factor *α* (TNF-α) kits were purchased from MULTI SCIENCES (LIANKE) BIOTECH Co., Ltd. (Hangzhou, China). Detergent Compatible Bradford Protein Assay Kit was purchased from Beyotime Biotechnology (Shanghai, China).

### 2.2 Preparation and optimization of ISL@NPs

ISL were loaded in zein/Cas complex NPs by anti-solvent precipitation method. The optimal ISL@NPs formula was optimized by single factor design based on the drug encapsulation efficiency (DEE) as the evaluation index. The feeding dosage of zein, Cas and ISL, as well as the ratio of organic/aqueous phase were optimize. Specifically, a certain amount of ISL and zein were co-dissolved in ethanol (80%, v/v), and then dropwise added into pure water containing Cas. The dripping and mixture process were carried out under stirring at 500 r/min for 2 h at 40°C. The residual ethanol in nano-suspension were removed and filtered with the microporous membrane (0.8μm, water). The amount of ISL was varied from 3 to 6 mg. The amount of zein was varied from 10 to 40 mg. The amount of Cas was varied from 10 to 40 mg. The volume ratio of ethanol/aqueous phase includes 1:10, 1:20, 2:10, and 2:20, responsively.

### 2.3 Characterization of ISL@NPs

The particle diameter and zeta potential of ISL@NPs were determined using a dynamic light scattering (DLS, Litesizer 500, Anton Paar, Austria) at room temperature. The morphology of ISL@NPs was obtained using Transmission Electron Microscope (TEM, Hitachi-7800, Japan) at an accelerating voltage of 80.0 kV.

The drug encapsulation efficiency (DEE) and drug loading efficiency (DLE) of ISL@NPs were detected by high-performance liquid chromatography (HPLC) (LC-2030C, SHIMADZU, Japan). In brief, ISL@NPs suspension was diluted three times with methanol and then extracted by ultrasonic in a water bath for 20 min. After filtration by a 0.22 μm microporous membrane, 10 μl of the filtrate was injected into HPLC. The content of isoliquiritigenin was detected using a C18 column (250 mm × 4.6 mm, 5 μm) at the maximum absorption wavelength of 372 nm. The mobile phase was methanol-water (60:40, v/v). The flow rate was 1.0 ml/min.

Drug encapsulation efficiency (DEE) and drug loading efficiency (DLE) of ISL@NPs were calculated by the following formulas (Xian et al., 2021).

$$\text{DEE}\left(\text{\%}\right)=\frac{\text{Amount of ISL loaded }}{\text{Amount of ISL added }}\times 100\text{\%}$$ (1)

$$\text{DLE}\left(\text{\%}\right)=\frac{\text{Amount of ISL loaded}}{\text{Total amount of ISL}@\text{NPs harvested}}\times 100\text{\%}$$ (2)

The storage stability of ISL@NPs at 4°C for 10 consecutive days was evaluated using particle diameter as the index. Then to investigate whether ISL@NPs fit as the oral administration system, we evaluated the particle diameter change of ISL@NPs in simulated gastric fluid (SGF, pH 1.5), simulated small intestine fluid (SIF, pH 6.8), and simulated colonic fluid (SCF, pH 7.8), respectively. After incubating at 37°C for 2 h, the appearances and the particle diameter changes were measured.

To investigate the encapsulation of ISL in zein/Cas NPs, Fourier transformation infrared spectroscopy (FT-IR) and X-ray diffraction (XRD) were employed. Briefly, the free pure ingredients (zein, Cas, and ISL), ISL@NPs and Blank NPs, as well as the physical mixture powder of ISL and Blank NPs, were investigated by FT-IR (Nicolet iS5, United States). The main steps were to grind the sample and potassium bromide in a particular proportion, press into a tablet, and then scan 32 times under the condition of the wavenumber of 400-4000cm^−1^ and resolution of 4cm^−1^. Likewise, these samples were determined using XRD patterns at room temperature (25°C), which were recorded using a Bruker D8 X-ray diffractometer (Brucker D8, Germany), range from 5° to 70° in continuous mode using a step size of 0.02° and 0.1s/step test speed (Zhang et al., 2022b).

### 2.4 Cellular uptake of C6-labeled NPs *in vitro*

Mouse leukemia monocyte macrophages (Raw 264.7) and human normal colonic epithelial cells (NCM 460) were maintained in Dulbecco’s Modified Eagle Medium (DMEM) (4.5 g/L glucose) and RAPI-1640 complete medium, supplemented with 10% heat-inactivated fetal bovine serum (Gibco, United States) and 1% penicillin-streptomycin. Both Raw 264.7 and NCM 460 cells were obtained from Shanghai Gaining Biological Technology Co., Ltd. (Shanghai, China) and incubated in an incubator at 37°C with 5% CO_2_.

Briefly, NCM460 and Raw264.7 cells were respectively seeded on confocal dishes (glass diameter 20 mm). The fluorescence dye C6 was loaded in zein/Cas NPs to label NPs. After 12 h incubation for cell adherence, free C6 and C6@NPs with 100 ng/ml of C6 were used to incubate with these cells for 4 h. These cells were fixed with paraformaldehyde for 15 min and then stained with Hoechst 33,342 (10 μg/ml) for 15 min. Cells were washed by cold PBS for 3 times, and then a drop of anti-fluorescence quenching agent was added. Laser scanning confocal microscope (CLSM) was employed to observe the internalization of NPs in cells.

### 2.5 Animal care

Institute of Cancer Research (ICR) mice (body weight, 22–25 g) were purchased from SPF Biotechnology Co., Ltd. (Beijing, China) and acclimatized 3 days before experimentation. Mice were fed in captivity under standard laboratory conditions (25 ± 2°C, 50 ± 5% RH). Mice had free access to standard rat chow pellets and water *ad libitum*. The experimental protocol was approved by the Institutional Animal Ethical Committee (IAEC) of Chengdu University of Traditional Chinese Medicine.

### 2.6 Ulcerative colitis model

Acute ulcerative colitis mice model induced by oral administration of 3% DSS (w/v) for 3 days (molecular weight: 36−50 kDa). After cessation of DSS feeding, mice with some representative symptoms like body weight loss and hematochezia were selected as UC model mice for the further investigation.

### 2.7 *In vivo* colon biodistribution of DiR-labeled NPs

DiR as a near-infrared fluorescent probe was primarily used to label zein/Cas NPs. Free DiR and DiR@NPs (2.5 mg/kg) were orally administered to DSS-induced UC mice for once. After 3, 6, 12, and 24 h post-administration, the whole mice were imaged by IVIS^®^ Spectrum *In Vivo* Imaging System (PerkinElmer, United States). Meanwhile, the colon tissues were excised at these each time-point, washed and imaged using the *In Vivo* Imaging System. The average amount of fluorescence was analyzed by the IVIS software.

### 2.8 Penetration of C6-labeled NPs in the colon of ulcerative colitis mice

To further investigate the tissue permeability of zein/Cas NPs to colonic epithelial sites *in vivo*, either the fluorescent probe C6 or C6-labeled NPs (5 mg/kg) were administered to DSS-induced UC mice, respectively. After 12 h, mice were sacrificed. The colon tissues were collected, fixed with 4% paraformaldehyde and dehydrated, then dropped into the OCT embedding agent and frozen. After the frozen sections with a thickness of 6 μm were rewarmed, they were stained with DAPI for 10 min at room temperature, then washed with PBS for 3 times with 5 min each time. Finally, these penetrated C6 in colon tissue were captured using CLSM (TSC SP8 SR, Leica, Germany).

### 2.9 Anti-ulcerative colitis effects of ISL@NPs in DSS-induced mice

Male ICR mice were randomly divided into five groups: normal control, UC model group, free ISL-treated group (30 mg/kg of ISL), ISL@NPs-treated group (15 mg/kg and 30 mg/kg of ISL, respectively), with six mice per group. Mice in normal control group received free access to autoclaved water, and the other groups were given for 3% DSS (w/v) for 10 consecutive days. At day 4, various ISL formulations were orally administrated. The control mice were given the equal volume of saline. During the whole experiment, the DAI score was used to evaluate the clinical progression of colitis, combining the weight loss, stool consistency, and fecal bleeding. (Gupta et al., 2015; Shi et al., 2020).

Twenty-four hours after the last treatment, mice were sacrificed by isoflurane euthanasia. The colon tissues from the ileocecal junction to the anal edge were harvested, photographed and measured the colon length. The colon tissue was immersed in 4% paraformaldehyde for 24 h for fixation, then transparent, immersed in wax, embedded, and cut into 4 μm thick wax slices. The colon tissue sections were stained with hematoxylin/eosin (H&E) and periodic acid schiff (PAS), respectively. Furthermore, in order to observe the infiltration of neutrophils and macrophages in colon tissue, the paraffin colonic sections were de-waxed and hydrated followed by antigen retrieval to recover protein structure. After PBS washing, the sections were incubated with primary anti-myeloperoxidase (1:100) overnight. And then the secondary antibody FITC-labeled goat anti-rabbit with green fluorescence, and AF647(F4/80) antibody with red fluorescence were incubated with sections to label neutrophils and macrophages, respectively. Finally, the nuclei were stained with DAPI and sealed with the anti-fluorescence attenuator after washing with PBS. The images were caught by an Olympus microscope.

Colon tissues were weighed and homogenized in cold PBS, followed by centrifugation at 8,000 r/min for 10 min at 4°C. And then, the supernatant was collected to analyze the levels of TNF-α and IL-6 by enzyme-linked immunosorbent assay (ELISA) kits (MULTI SCIENCES, China), according to the manufacturer’s instructions. Total protein concentration was measured using the Detergent Compatible Bradford Protein Assay Kit (Beyotime, China).

### 2.10 Statistical analysis

Statistical comparisons of all data between multiple groups were performed using one-way analysis of variance (ANOVA) followed by the Tukey test in GraphPad Prism 8.0.2 (La Jolla, California) at a significance level of *p* < 0.05. Data is represented as the mean ± standard deviation (SD).

## 3 Results

### 3.1 Single-factor experimental results

Based on the previous study, some main key parameters in anti-solvent nano-precipitation method including the feeding dosages of zein, Cas, and ISL, as well as the volume ratio of organic phase and water phase, were optimized. We found that the most significant influence factor was DEE, instead of particle diameter. These parameters at different levels exhibited the different DEE of ISL@NPs. Based on the DEE result, the optimized condition was as followings: the feeding amount of zein, Cas, and ISL was 20 mg, 20 mg, and 4 mg, respectively; the organic phase and water phase was 2 ml 80% ethanol and 20 ml pure water, respectively. Finally, under these optimal conditions, the final average encapsulation efficiency of ISL@NPs was 96.15 ± 1.53%.

### 3.2 Characterization of ISL@NPs

**FIGURE 1:**
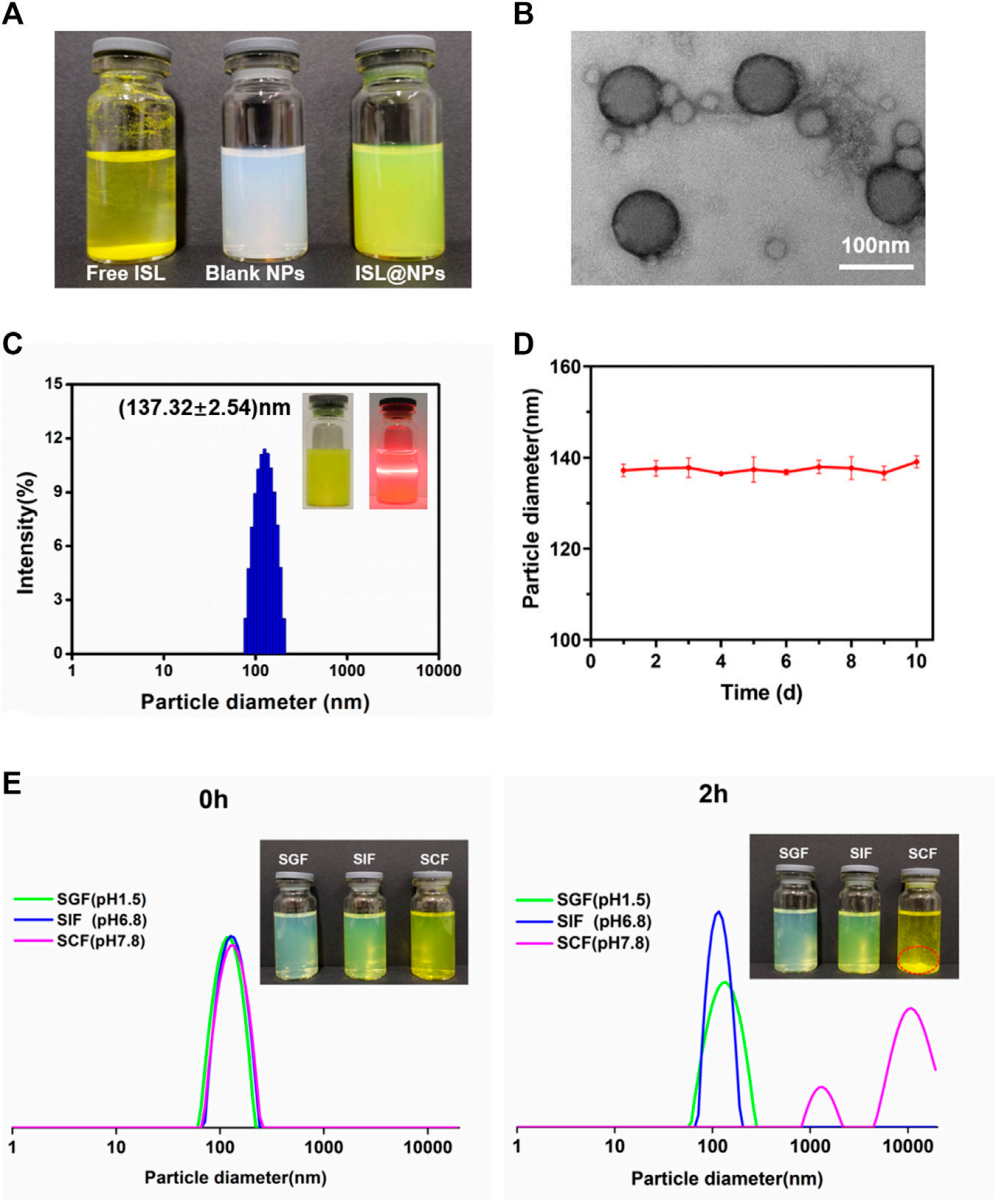
Characterization of ISL@NPs. **(A)** The appearance of homogeneous suspension of ISL@NPs, in comparison to the insolubility of free ISL in water. **(B)** TEM image of ISL@NPs. **(C)** Particle diameter distribution of ISL@NPs by DLS measurement. Tyndall effect could be observed in ISL@NPs. **(D)** The storage stability of ISL@NPs at 4°C for 10 d **(E)** ISL@NPs exhibited pH-responsive instability in SCF (pH 7.8), in comparison to the stability in SGF (pH 1.5) and SIF (pH 6.8).

Unlike free ISL, which was insoluble in water, precipitated at the bottom of the vial, the prepared blank and drug-loaded nanoparticles were evenly suspended in the medium without visible precipitation (Figure 1A). From the TEM image in Figure 1B, it could be seen that the ISL@NPs presented a complete and smooth spherical shape. As shown in Table 1 and Figure 1C, the average particle diameter of the ISL@NPs was (137.32 ± 2.54) nm with an average polydispersity index less than 0.3, and the average potential of ISL@NPs was (−37.81 ± 0.66) mV, showing strong electronegativity. The particle diameter of blank nanoparticles was smaller than that of drug-loaded nanoparticles. ISL@NPs had a uniform particle diameter and a high absolute value of negative potential, which meant that the nanoparticles could well combine with the positively charged proteins of colon epithelial cells and avoid the aggregation of nanoparticles, making the nanoparticles have satisfactory stability (Luo et al., 2020). This result was further confirmed in the stability study. In addition, quantification of the loaded ISL via HPLC revealed that the encapsulation efficiency and loading efficiency of ISL@NPs were 96.15 ± 1.53% and 9.39 ± 0.26% (Table 1).

**TABLE 1:** Physicochemical characteristics of blank NPs and ISL@NPs (*n* = 3).

Sample	Particle diameter (nm)	PDI (%)	Zeta potential (mV)	DEE (%)	DLE (%)
Blank NPs	126.33 ± 2.67	0.14 ± 0.01	−27.00 ± 0.35	—	—
ISL@NPs	137.32 ± 2.54	0.12 ± 0.01	−37.81 ± 0.66	96.15 ± 1.53	9.39 ± 0.26

Next, we used the particle diameter and polydispersity index as indicators to prove the stability of NPs further. The results showed that the particle diameter of ISL@NPs had almost no change during the storage process of 10 days (Figure 1D). The harsh microenvironment of the gastrointestinal tract was the biggest challenge for oral drug delivery systems. The stability of nanoparticles in the gastrointestinal tract determined whether the drug could reach the colon, and the gastrointestinal stability of nanoparticles also guided us to further develop animal studies. As shown in Figure 1E, at 0h, the particle diameter of ISL@NPs in SGF (PDI = 0.158 ± 0.014), SIF (PDI = 0.125 ± 0.070), and SCF (PDI = 0.156 ± 0.036) was uniform, which was the same as that of freshly prepared nanoparticles. After two hours of incubation, the particle diameter of ISL@NPs remained stable in SGF and SIF. While in SCF, the aggregation of ISL@NPs resulted in macroscopic precipitates with multiple particle diameter peaks. The results showed that ISL@NPs had good stability in SGF and SIF, and could avoid the premature release of ISL in the stomach and small intestine, and it was easy to swell and aggregate in the colon for drug release.

### 3.3 FT-IR and XRD

**FIGURE 2:**
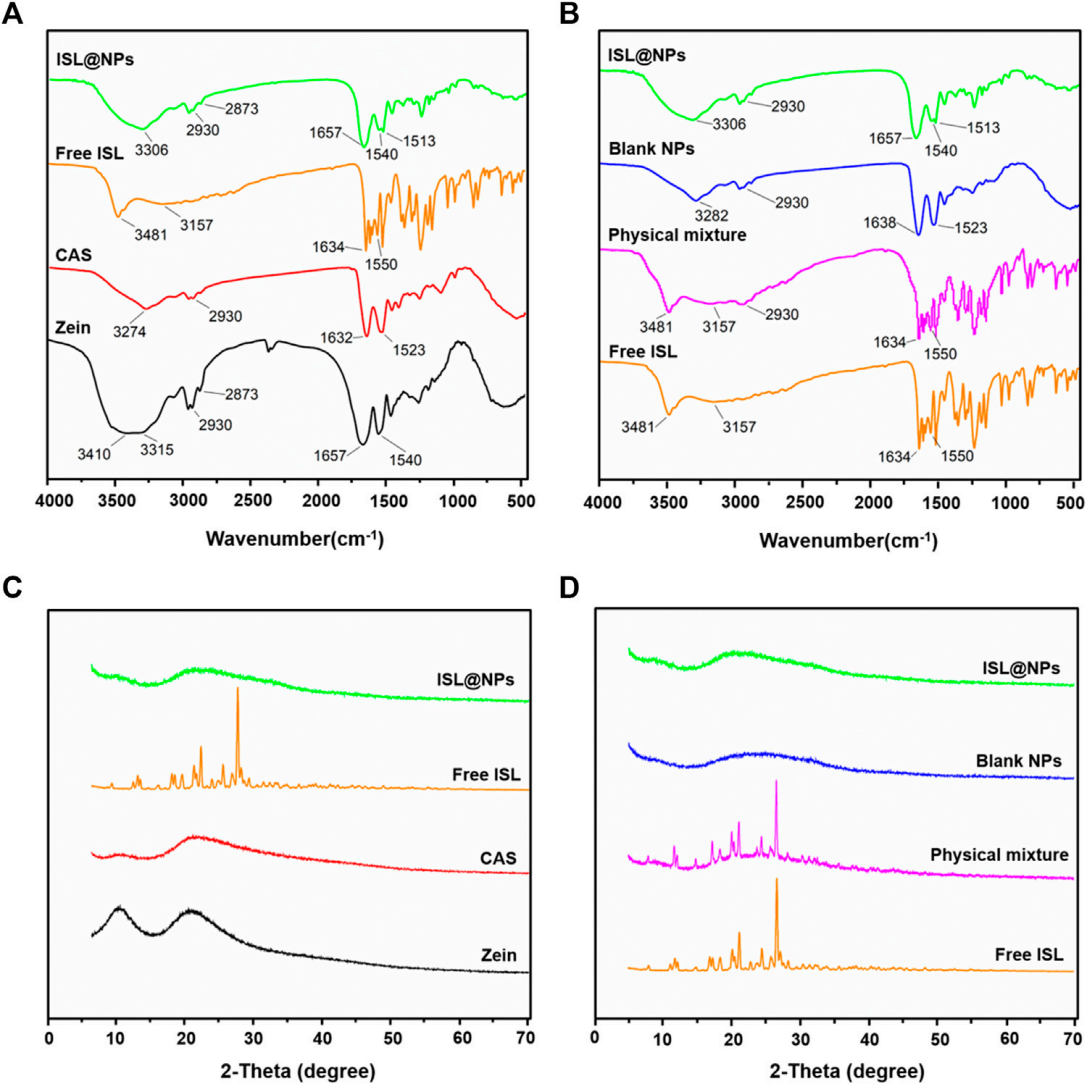
FT-IR **(A-B)** and XRD **(C-D)** were implemented to characterize ISL@NPs.

The potential interactions between zein, Cas, and ISL were investigated using FT-IR. The infrared spectrum was shown in Figure 2A, the characteristic peaks of zein appear at 3,410 cm^−1^ and 3,315 cm^−1^, the characteristic peak of Cas appeared at 3,274 cm^−1^, and the strong and broad peaks from 3,100 cm^−1^ to 3500cm^−1^ represent the -OH stretching vibration of hydroxyl-bound water. (Chang et al., 2017). The characteristic peaks at 2930cm^−1^ and 2,873 cm^−1^ of zein belonged to the stretching of the COOH carboxylic acid group, and the characteristic peak at 2930cm^−1^ could also be observed in Cas. In addition, zein showed two prominent peaks of 1,656 cm^−1^ and 1,540 cm^−1^ in the range of 1,500–1700 cm^−1^ (Yuan et al., 2020; Shirmohammadli et al., 2021). Within the same regional range, the characteristic peaks of Cas were at 1,632 cm^−1^ and 1,523 cm^−1^ (Li et al., 2021a), which represented the amide I band and amide II band, respectively. The amide I band was related to the stretching of C=O, and the absorption peak of amide II was due to C-N stretching and the bending vibration of the N-H group (Chen et al., 2022). Interestingly, a sharper peak with stronger intensity was observed at 2,930 cm^−1^, which may be attributed to the better hydrophobicity of zein than Cas (Chang et al., 2017; Dai et al., 2018a). The absorption band of free ISL near wavenumber 3481 cm^−1^ was the stretching vibration absorption of hydroxyl (−OH), and the wavenumber at 3,157 cm^−1^ was the stretching vibration absorption of carbon-hydrogen bond (=C-H). The wavenumber of 1,634 cm^−1^ was the absorption peak caused by the stretching vibration of carbon-carbon double bond (C=C), (Zhao et al., 2021), the characteristic absorption peak of the aromatic nucleus at 1,550 cm^−1^ (Li et al., 2015). It can be seen from the figure that in ISL@NPs, the characteristic peaks of free ISL are completely covered, indicating that zein and Cas have a good entrapment effect on ISL.

As shown in Figure 2B, for the three physical mixtures, characteristic peaks of free ISL 3481 cm^−1^, 3,157 cm^−1^, 1,634 cm^−1^, and 1,550 cm^−1^ could be clearly observed. However, the spectrum of ISL@NPs was similar to that of Blank NPs, and no characteristic peaks of ISL appeared, indicating that ISL was not physically entrapped in zein and Cas. It was worth noting that after the formation of nanoparticles, the characteristic peaks of the OH stretching vibration of zein and Cas were both shifted. In ISL@NPs, the characteristic peaks changed from 3,315 cm to 1 to 3,306 cm^−1^. In Blank NPs, the characteristic peak of Cas changed from 3,274 cm^−1^ to 3,282 cm^−1^, indicating the formation of hydrogen bonds among the ingredients. Meanwhile, in the nanoparticles, the amide I and amide II bands of zein and Cas were also shifted, indicating that the formation of ISL @Zein NPs and Blank @Zein NPs was related to electrostatic interactions (Luo et al., 2011; Chen et al., 2022).

As shown in Figure 2C, the diffraction peaks of zein and Cas showed two flat peaks at 9.276° and 19.390°, 9.550° and 20.120°, respectively, both without sharp characteristic peaks, indicating that zein and Cas were in an amorphous state (Dai et al., 2018a; Shirmohammadli et al., 2021; Chen et al., 2022). The peaks of free ISL at 2 theta values of 7.959°, 11.121°, 11.835°, 12.182°, 14.896°, 17.304°, 18.423°, 20.181°, 21.221°, 22.833°, 24.486°, 25.812°, and 26.690° proved that ISL was crystalline (Qiao et al., 2020). However, ISL@NPs showed a large amorphous peak at 20.201°. As shown in Figure 2D, for the physical mixture, the diffraction pattern was just the superposition of the three patterns of ISL crystals and zein and Cas, indicating that no inclusion complexes were formed between the three compounds and still maintained their original physical properties (Li et al., 2015). The patterns of ISL@NPs were similar to Blank NPs and did not show any crystallinity peaks for ISL. In conclusion, the experimental XRD crystallization results showed that the nanoparticles had successfully encapsulated ISL.

### 3.4 Cellular uptake *in vitro*

**FIGURE 3:**
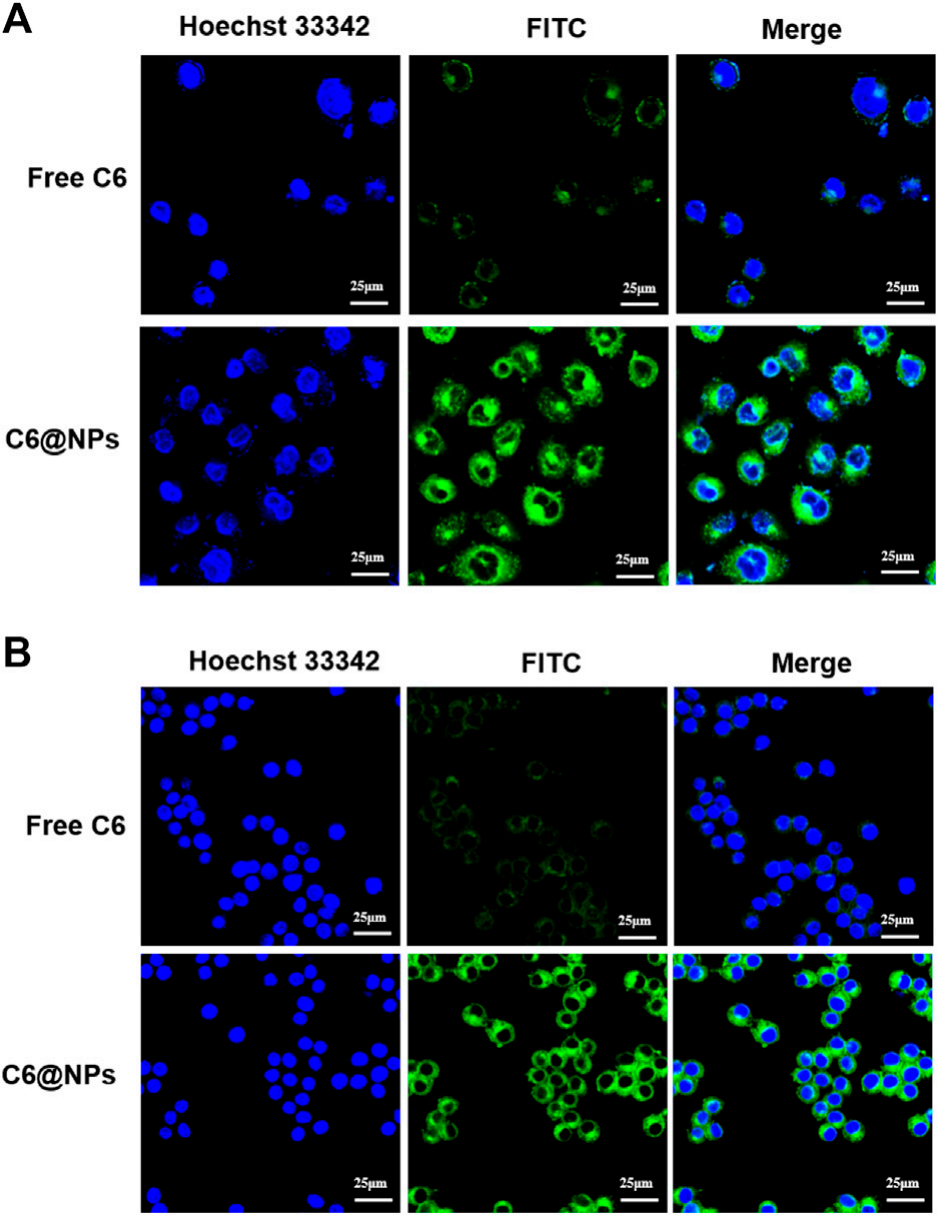
With the fluorescence probe C6 used to label zein-Casein NPs, the cellular uptake advantages of zein-Casein NPs in NCM 460 cells **(A)** and Raw 264.7 cells **(B)** were investigated by CLSM, after 4 h incubation with the C6 concentration of 100 ng/ml.

CLSM is a standard qualitative method for observing cellular uptake. As can be seen from CLSM images in Figure 3, the green fluorescence of free C6 in NCM 460 cells (Figure 3A) and Raw 264.7 macrophages (Figure 3B) detected was poor and barely visible after 4 h of administration, whereas C6@NPs showed bright fluorescence in both types of cells. The cellular uptake results showed that the C6@NPs could enter intestinal epithelial cells and macrophages more efficiently than free C6, which revealed retention and particular cellular targeting.

### 3.5 *In vivo* colon biodistribution of DiR-labeled NPs

**FIGURE 4:**
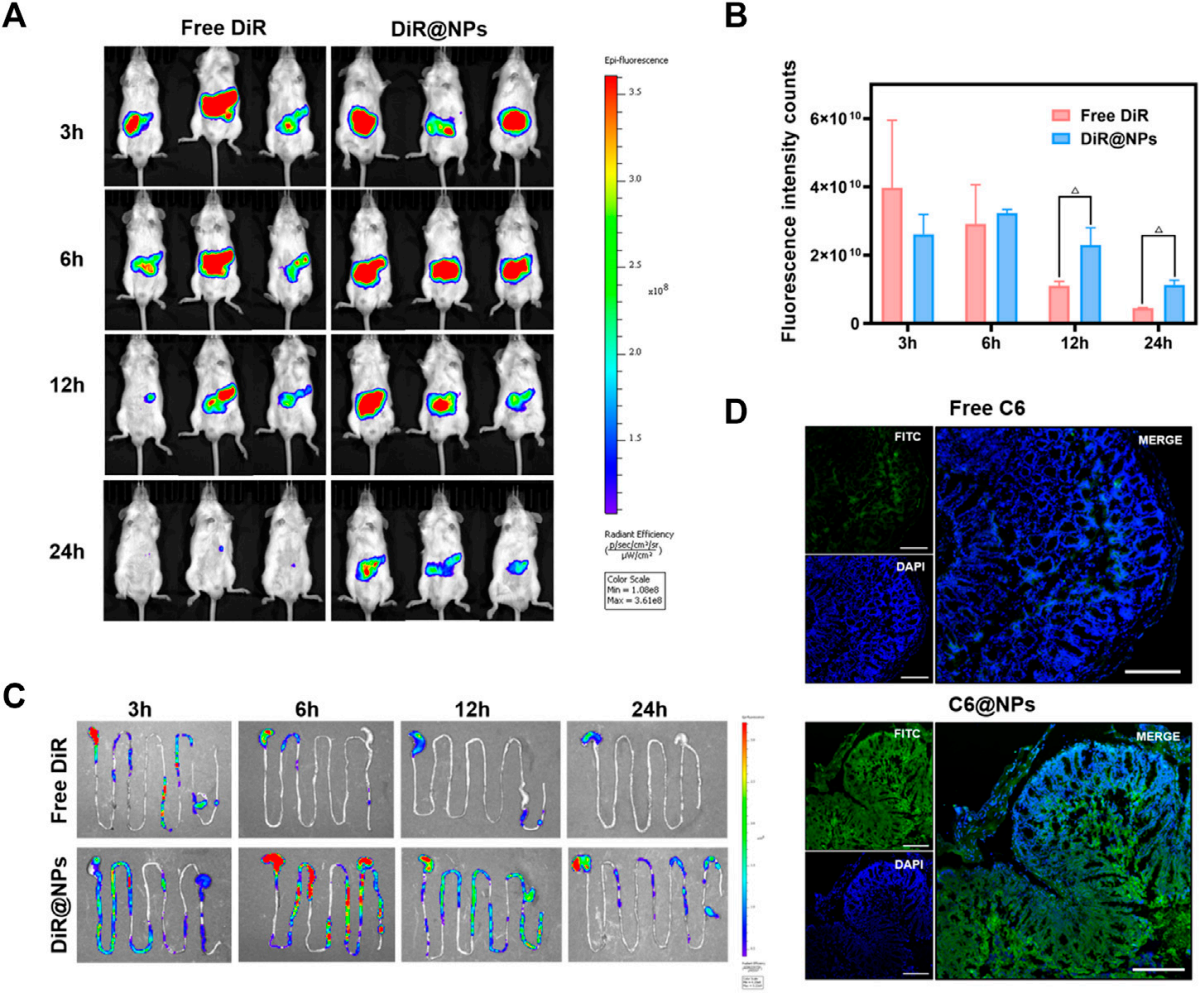
After the near-infrared fluorescent dye DiR and C6 loaded into zein-Casein NPs, the advantages of zein-Casein NPs on colon-specific accumulation and tissue penetration were observed. **(A)** Fluorescence images of mice at 3, 6, 12, and 24 h after oral administration of free DiR and DiR@NPs. **(B)** Histogram of fluorescence intensity in mice at 3, 6, 12, and 24 h after oral administration of free DiR and DiR@NPs (n = 3, ^Δ^ *p* < 0.05). **(C)** Fluorescence images of the gastrointestinal tract of mice at 3, 6, 12, and 24 h after oral administration of free DiR and DiR@NPs. **(D)** Fluorescence images of frozen sections after oral administration of free C6 and C6@NPs for 12 h, green fluorescence represented C6, and blue fluorescence represented the location of the cell nucleus (scale bar = 200 μm).

*In vivo* distribution analysis is an essential step to study drug retention or targeting at the lesion site when evaluating the oral nano drug delivery system (Pu et al., 2021). DiR and DiR@NPs were prepared using fluorescent dye DiR instead of non-fluorescent ISL. Images were obtained using IVIS to assess the specific delivery potential of the formulation at the colonic site. The results were shown in Figures 4A–C, the average fluorescence intensity across the two groups did not show a significant difference after gavage for 3 h, and most of the fluorescence stayed in the stomach. After 6 h of administration, most free DiR was quickly metabolized through the colon, and the mean fluorescence intensity decreased significantly. However, the DiR@NPs showed stronger fluorescence intensity than at 3 h, as shown in Figure 4C, which may reflect the accumulation of the DiR@NPs in the colon after 6 h of gavage. After 12 h of administration, the fluorescence signal of the gastrointestinal tract of the mice in the free group declined sharply. In contrast, the average fluorescence intensity of the mice in the NPs group only showed a slight decrease, which was significantly different from that of the free DiR group (*p* < 0.05) and continued to the endpoint (24 h). These results further suggested that the shell formed by the zein and Cas protected ISL well, prevented the sudden release of the drug in the stomach and small intestine, and delivered them specifically to the inflammatory site of the colon.

### 3.6 Penetration of C6-labeled NPs in the colon of ulcerative colitis mice

We replaced ISL with the fluorescent dye C6 to verify whether ISL@NPs could deliver and penetrate ISL into the inflammatory colon tissue. UC mice were given different C6 preparations for 12 h to obtain frozen sections of the inflamed colon, and DAPI was used for nuclear staining (blue fluorescence) of the frozen sections, as shown in Figure 4D. Free C6 (green fluorescence) was rarely distributed in colon tissue. However, the green fluorescence of C6@NPs was strong and penetrated into colonic epithelial tissue. These results suggest that C6@NPs effectively transport C6 across the intestinal barrier into colon tissues. Zein and Cas had a protective effect on C6 before the NPs reached the colon.

### 3.7 Anti-ulcerative colitis effects of ISL@NPs in DSS-induced mice

#### 3.7.1 Colon length, body weight changes, and diarrhea scores

**FIGURE 5:**
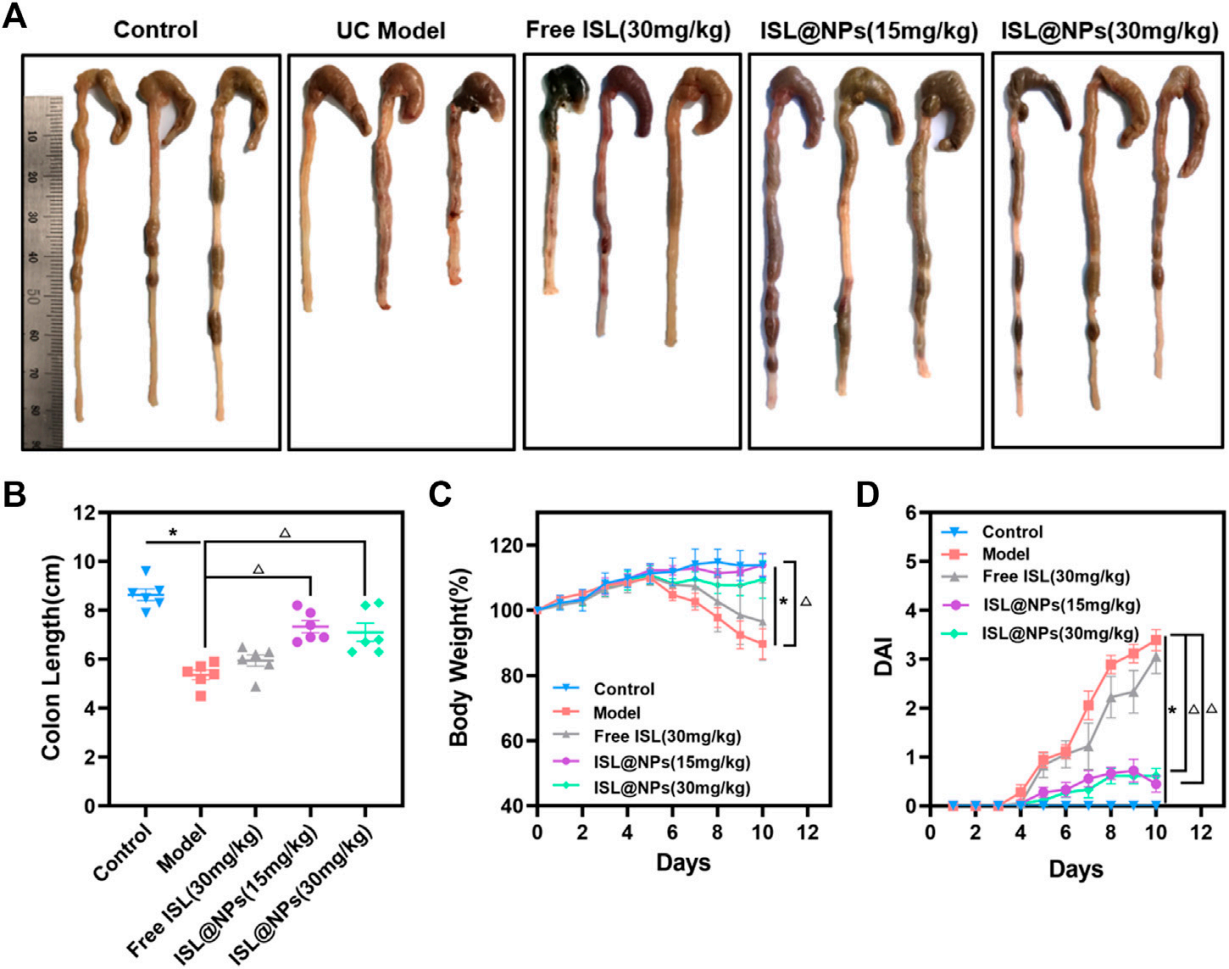
*In vivo* therapeutic efficacy of ISL@NPs on DSS-induced UC mice after 10 days administration. **(A)** Representative colon photographs of mice in different groups at day 10. **(B)** Quantitative analysis of colon length in different groups at day 10. The body weight curve **(C)** and the curves of DAI score **(D)** of mice in different groups during the whole experimental period. ^*^ *p* < 0.05 (model group vs. control group), ^Δ^ *p* < 0.05 (model group vs. free ISL and ISL@NPs groups).

The main symptoms of ulcerative colitis were diarrhea, hematochezia, and weight loss (Wang et al., 2021). Therefore, DAI score, weight, and colonic length changes were used as evaluation indexes of disease severity in mice. We compared the UC model group with the control group and the ISL-related groups, as shown in Figure 5. It can be observed in Figures 5A,B that the colon of mice in the UC model group was significantly swollen and congested compared with that in the control group. The length of the colon of mice in the UC model group was shorter (5.37 cm ± 0.49 cm) than that in the control group (8.63 cm ± 0.57 cm), and the severity of the free ISL group was similar to the model group. However, the colon lengths of the NPs groups at 15 mg/kg of ISL and 30 mg/kg of ISL reached (7.33 cm ± 0.61 cm) and (7.10 cm ± 0.91 cm), respectively. In terms of colon length, the ISL preparation groups were significantly different from the free ISL group and the model group. Ameliorated the disease state of shortened colon in UC mice.

One of the major signs of the colitis phenotype is a decrease in body weight (Xu et al., 2022). We recorded the changes in the body weight of mice in each group. As shown in Figure 5C, compared with the UC model group, the weight of the ISL@NPs treated mice recovered well, and the 15 mg/kg of ISL NPs group showed a better recovery state than the 30 mg/kg of ISL NPs group. In addition, DAI scores (Figure 5D) showed that severe diarrhea and hematochezia occurred in the UC model group, while DAI scores were significantly reduced in the ISL@NPs group. In conclusion, the results of UC-related indicators, such as colon length, body weight, and DAI scores indicated that treatment with ISL@NPs could significantly improve the symptoms of DSS-induced ulcerative colitis, and the efficacy was much better than free ISL.

#### 3.7.2 Histopathological evaluation of colitis severity

**FIGURE 6:**
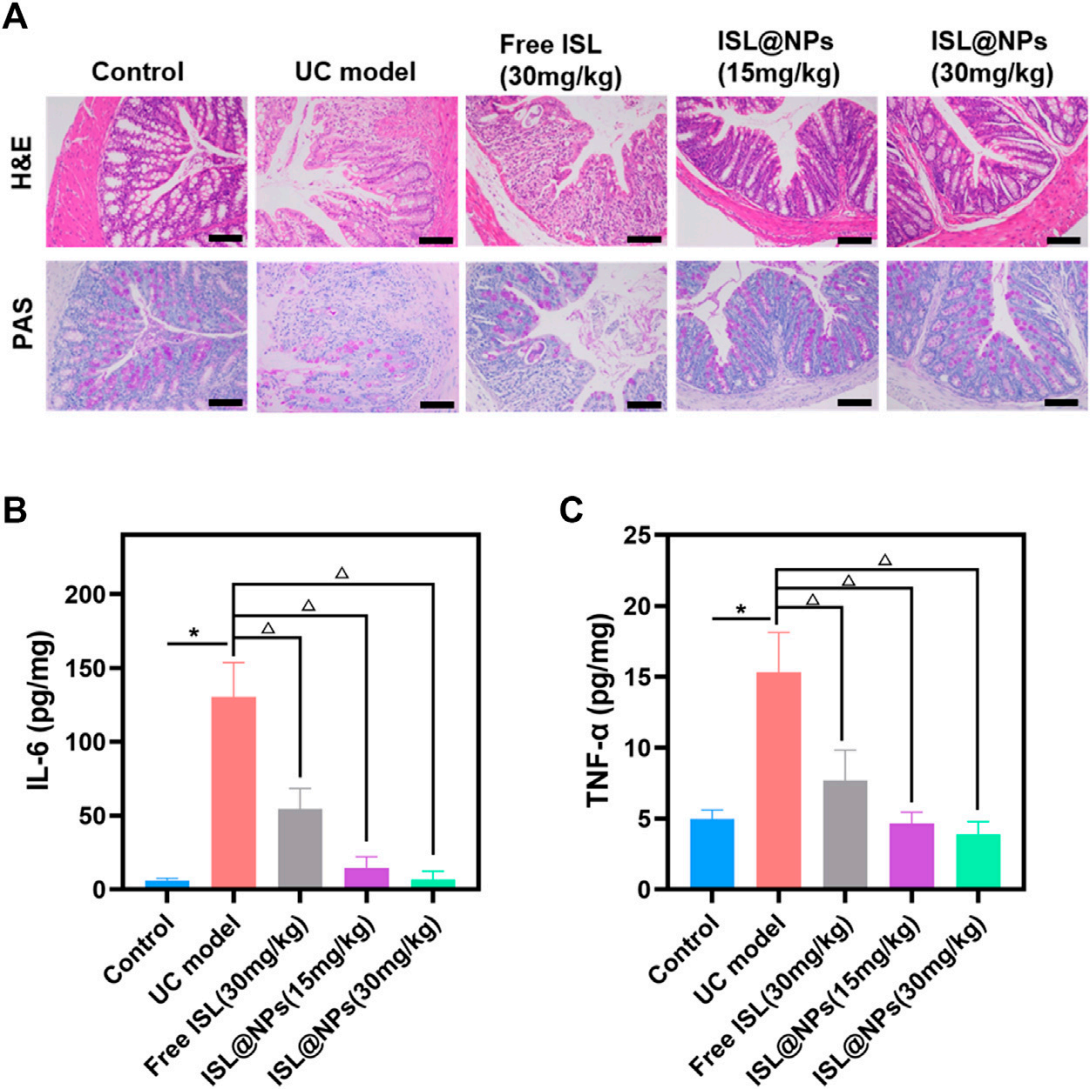
Histopathological evaluation and inflammatory factor determination. **(A)** Histopathological evaluation by H&E and PAS staining for different groups. Scale bar = 100 μm. The levels of IL-6 **(B)** and TNF-α **(C)** in colon tissue of mice at day 10. ^*^ *p* < 0.05 (model group vs. control group), ^Δ^ *p* < 0.05 (model group vs. free ISL and ISL@NPs groups).

The severity of colitis was histologically evaluated by H&E staining and PAS staining of colon tissue. H&E staining and PAS staining reflected the inflammatory damage of colon tissue and the situation of colonic mucus goblet cells, respectively (Wang et al., 2021). The representative picture was shown in Figure 6A, the colonic tissue of the control mice showed no signs of inflammation or destruction of healthy tissue morphology. However, DSS-induced model group presented severe intestinal edema, damaged epithelial cells, massive loss of mucus-secreting goblet cells and crypts, and severe inflammatory cell infiltration. Compared with the model group, the inflammation of the mice in the ISL@NPs treatment groups got significant anesis, which was manifested by the reduction of inflammatory cell infiltration and the protection of the structural integrity of the colon, and the colon tissue morphology was similar to that of healthy mice. The staining results indicated an excellent therapeutic efficacy of ISL@NPs.

#### 3.7.3 Determination of IL-6 and TNF-α levels in colon tissues

IL-6 and TNF-α are pro-inflammatory factors expressed at high levels by M1 macrophages, and their levels reflect the infiltration of macrophages and are closely related to the development of inflammation (Zhang et al., 2020). The levels of pro-inflammatory cytokines IL-6 and TNF-α in colon tissues of all groups were determined by ELISA. As shown in Figures 6B,C, the levels of IL-6 and TNF-α were higher in the UC model by comparing it with the control group and ISL-related groups. (*p < 0.05; ^Δ^ *p* < 0.05). Free ISL revealed certain inhibitory effect on pro-inflammatory cytokines, but loading ISL into zein and Cas further enhanced the anti-inflammatory capacity of ISL. Treatment with ISL@NPs clearly reduced the pro-inflammatory cytokine levels and made them close to untreated control mice.

### 3.7.4 Infiltration of neutrophils and macrophages and in colon tissue

**FIGURE 7:**
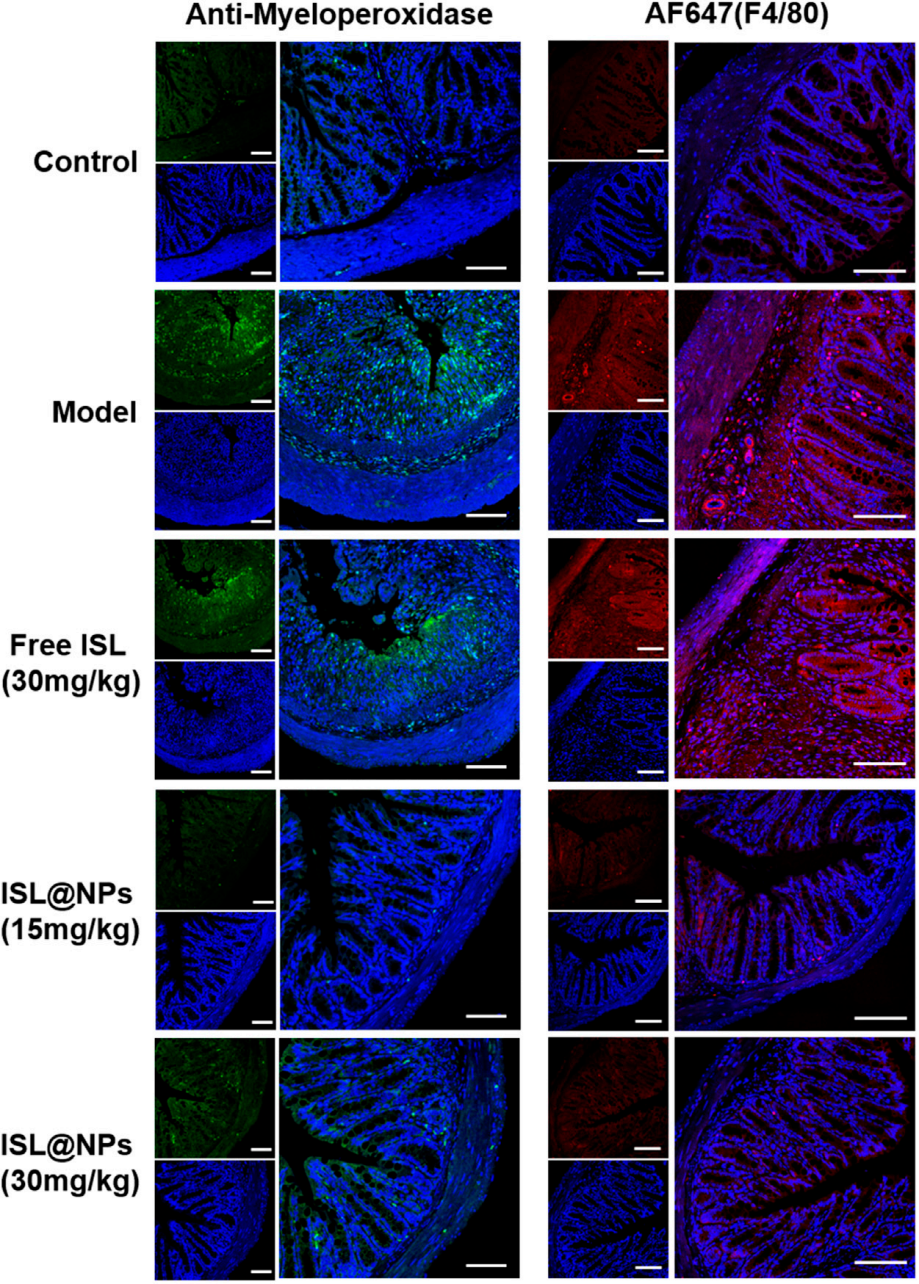
Immunofluorescence staining of neutrophils and macrophages in colon tissues of mice from different groups. The blue, green, and red fluorescence derived from the stained nuclei, neutrophils, and macrophages, respectively. Scale bar = 100 μm.

Anti-myeloperoxidase antibody was used to immunostain paraffin sections of colon tissue in each group. The green fluorescence of the antibody represented the number and distribution of neutrophils. As shown in Figure 7, compared with control mice, extensive neutrophil infiltration was observed in DSS-induced UC model and free ISL group. Notably, ISL@NPs could largely reduce neutrophil infiltration in the colonic mucosa of UC mice. Immunostaining of paraffin sections of tissues with Alexa Fluor 647 (AF 647) labeled F4/80 antibody showed the location of macrophages in red fluorescence, and increased infiltration of macrophages in colon tissues of UC model mice confirmed the severity of colitis. The effect of free ISL on the infiltration of inflammatory cells in colitis was not significant. However, both of the green and red fluorescence in colonic tissue of mice treated with ISL@NPs were rather weak, indicating a prominent reduction in the number of neutrophil macrophages compared with the UC model group and a noticeable recovery of inflammation. The above results were consistent with ELISA measurements of pro-inflammatory cytokines levels in colon tissues of each group.

## 4 Discussion

When administered orally, polymer carriers or polymer-coated formulation prevent premature drug release and degradation in the upper gastrointestinal tract. In addition, the polymer carrier can also promote the selective accumulation and controlled release of the drug at the site of colonic inflammation. A variety of polymers have been used as effective drug carriers, which can solve the problem of local specific drug delivery in the colon. Commonly used composite materials for colon-targeted drug delivery systems are mainly polysaccharide-based polymers (chitosan, dextran, pectin, hyaluronic acid and ethyl cellulose), polyester-based biodegradable polymers (PLGA and PLA), and acrylic-based polymers (eudragit and carbomer) (Dar et al., 2017).

Although various polymers have been demonstrated to possess unique advantages in colon-targeted drug delivery, because of the challenges posed by the harsh gastrointestinal environment for the use of carrier materials alone, an increasing number of studies have combined composite materials with different properties for synergistic drug delivery (Wang et al., 2022). Coating of chitosan nanoparticles with Eudragit FS 30D enabled sustained and pH-responsive colon specific drug delivery, and the combined application of chitosan and Eudragit FS 30D solved the problems of poor absorption and availability of curcumin (Raj et al., 2018). In addition, the biomimetic nano system prepared by coating the cell membrane can also well overcome the problem of premature ejaculation of drugs by a single polymer material under the influence of the gastrointestinal environment. Compared with synthetic materials, macrophage membrane has the best biocompatibility. Wrapping PLGA NPs with macrophage membrane could significantly increase the accumulation of drugs in the inflammatory colon tissue, increase the tissue retention time, and reduce the risk of systemic exposure (Li et al., 2021b).

ISL, as a chalcone compound, has a significant curative effect in anti-colitis and even related tumors. ISL can interfere with IBD by inhibiting the pro-inflammatory cytokine-induced inflammatory response. In TNF-α induced intestinal epithelial HT-29 cells, ISL induces the activity of histone deacetylase and inhibits the release of acetylated high-mobility group box 1, thereby inhibiting the expressions of IL-8, IL-1β, and COX-2 inflammatory factors (Chi et al., 2017). As previously describe, the imbalance of intestinal flora is closely related to the onset of colitis, and may even induce colorectal cancer. The study has found that ISL can reverse and reduce the abundance of pathogenic bacteria such as *escherichia coli* and *enterococcus*, and improve the level of probiotics, which might cooperate with intestinal flora to fight colorectal cancer related to colitis (Wu et al., 2016). Moreover, ISL could inactivate PGE2/PPARδ and IL-6/34 STAT3 signaling pathways, inhibit M2 macrophage polarization, and effectively inhibit the occurrence of colitis-related tumors (Zhao et al., 2014).

Zein is an amphiphilic protein with two-thirds lipophilic amino acid residues and one-third hydrophilic amino acid residues (Luo et al., 2013). Due to the interaction between the positively charged nanocarrier and the negatively charged intestinal mucosa, the cationic nano delivery system can be adhered to the mucosal surface of the inflamed tissue, increases the targeting and retention properties of the colitis drug delivery system, and enables the positively charged NPs to possess stronger cell uptake capability (Hua et al., 2015). However, studies have found that cationic NPs damages the integrity of the plasma membrane more obviously, and its mitochondria and lysosomes are damaged more strongly. The number of autophagosomes is more than that of anionic NPs, resulting in certain cytotoxicity. By contrast, the negatively charged anionic NPs have more reasonable broad-spectrum biological characteristics (Frohlich, 2012; Sadat et al., 2016). On the other hand, zein nanoparticles are easy to aggregate and show explosion effect, and the stability of zein nanoparticles solution became worse after dilution, and it is difficult to redissolve after freeze-drying, so there are some limitations in preparing nanoparticles by using zein alone (Luo et al., 2013; Li et al., 2018). In our study, we made full use of the positive charge of zein to increase the targeting and retention of the drug delivery system and the advantage of self-assembly to form a core-shell structure. At the same time, Cas was used to overcome the disadvantages of zein such as instability and easy aggregation. Owing to hydrogen bond and electrostatic interaction, the nano system formed by Zein and Cas had high dispersibility and stability, and could efficiently deliver ISL to the colon inflammation site, increase nano-cell uptake capacity (Figure 3) and tissue permeability (Figure 4D), and reduce the infiltration of inflammatory cells (Figure 7).

## 5 Conclusion

In this study, we used zein and Cas as carriers to encapsulate ISL and produced ISL@NPs by the anti-solvent method. Based on the protective effect of zein and Cas, the early release of ISL in the stomach and small intestine could be reduced to a certain extent, and the aggregation in the colon could be increased. Compared with free ISL, nanoparticles showed stronger uptake and longer-lasting retention in colonic inflammation sites. More importantly, such a natural oral colon specific nano drug delivery system could promote the repair of damaged intestinal mucosa in UC mice, inhibit the high expression of pro-inflammatory factors, alleviate the severity of the disease, and improve the quality of life of the mice. In summary, the study combines natural proteins from food with natural active ingredients from plants. As a healthy and efficient nano-drug delivery system, ISL@NPs provide a beneficial avenue for clinical application in UC treatment.

## Data Availability

The original contributions presented in the study are included in the article/Supplementary Material, further inquiries can be directed to the corresponding authors.
